# Molecular Biomarkers for Gestational Diabetes Mellitus

**DOI:** 10.3390/ijms19102926

**Published:** 2018-09-26

**Authors:** Stephanie Dias, Carmen Pheiffer, Yoonus Abrahams, Paul Rheeder, Sumaiya Adam

**Affiliations:** 1 Biomedical Research and Innovation Platform, South African Medical Research Council, P.O. Box 19070, Tygerberg, Cape Town 7505, South Africa; Stephanie.Dias@mrc.ac.za (S.D.); carmen.pheiffer@mrc.ac.za (C.P.); Yoonus.Abrahams@mrc.ac.za (Y.A.); 2 Department of Obstetrics and Gynecology, University of Pretoria, Private Bag X169, Pretoria 0001, South Africa; 3 Division of Medical Physiology, Faculty of Health Sciences, Stellenbosch University, P.O. Box 19063, Tygerberg, Cape Town 7505, South Africa; 4 Department of Internal Medicine, Faculty of Health Sciences, University of Pretoria, Private Bag X169, Pretoria 0001, South Africa; paul.rheeder@up.ac.za

**Keywords:** molecular biomarkers, gestational diabetes mellitus, DNA methylation, microRNAs, single-nucleotide polymorphism, genetic and epigenetic markers

## Abstract

Gestational diabetes mellitus (GDM) is a growing public health problem worldwide. The condition is associated with perinatal complications and an increased risk for future metabolic disease in both mothers and their offspring. In recent years, molecular biomarkers received considerable interest as screening tools for GDM. The purpose of this review is to provide an overview of the current status of single-nucleotide polymorphisms (SNPs), DNA methylation, and microRNAs as biomarkers for GDM. PubMed, Scopus, and Web of Science were searched for articles published between January 1990 and August 2018. The search terms included “gestational diabetes mellitus”, “blood”, “single-nucleotide polymorphism (SNP)”, “DNA methylation”, and “microRNAs”, including corresponding synonyms and associated terms for each word. This review updates current knowledge of the candidacy of these molecular biomarkers for GDM with recommendations for future research avenues.

## 1. Introduction

Gestational diabetes mellitus (GDM) is defined as glucose intolerance with onset or first recognition during pregnancy [1]. The prevalence of GDM is increasing worldwide, with approximately 14% of pregnancies affected by GDM [2]. The condition is associated with perinatal complications and an increased risk for future metabolic disease in both mothers and their offspring. The oral glucose tolerance test (OGTT) is considered the gold standard for the diagnosis of GDM [3]. However, the test is cumbersome to conduct, requires fasting and multiple blood draws, and its association with nausea and vomiting leads to decreased patient compliance. Furthermore, the OGTT is conducted between 24–28 weeks of gestation [3], presenting a small window of opportunity to implement interventions to improve pregnancy outcomes. Earlier detection of GDM may lead to improved management, possibly preventing pregnancy complications. Thus, the identification of sensitive and specific biomarkers, which may offer potential for risk prediction and intervention strategies, became a major focus in GDM research. Several studies provided evidence for a genetic predisposition to GDM [4], while gene–environment interactions could explain the population-specific variation in GDM prevalence [5]. Consequently, single-nucleotide polymorphisms (SNPs) and epigenetic mechanisms are widely explored as molecular biomarkers for GDM.

The purpose of this review is to provide an overview of the current status of SNPs and the two most commonly investigated epigenetic mechanisms, DNA methylation and microRNAs (miRNAs), as molecular biomarkers for GDM. Three major databases, PubMed, Scopus, and Web of Science, were searched for studies published between January 1990 and August 2018 that investigated SNPs, DNA methylation, and miRNAs in the blood of women with GDM. Blood was selected as it is easily accessible as part of routine antenatal care. The search terms included “gestational diabetes mellitus”, “blood”, “single-nucleotide polymorphism (SNP)”, “DNA methylation”, and “microRNAs”, including corresponding synonyms and associated terms for each word (Appendix A, Table A1). Articles were selected if they reported case-control studies that investigated GDM in association with SNPs, DNA methylation, or miRNAs in maternal blood, plasma, or serum, and were conducted in humans. This review begins with an overview of GDM, followed by a brief description of the characteristics of ideal biomarkers. Thereafter, studies profiling SNPs and DNA methylation in whole blood, and miRNAs in whole blood, plasma, or serum of women with GDM are summarized, and the limitations of these molecular biomarkers are discussed. Finally, the current status of GDM biomarkers is discussed, along with recommendations for future research. 

## 2. Overview of Gestational Diabetes Mellitus

The exact mechanism underlying the development of GDM is not completely understood; however, it is speculated that women who develop GDM are unable to meet the increasing demand for insulin production during pregnancy [6]. GDM is associated with an increased risk of short- and long-term pregnancy complications. Women with GDM have a higher risk of pre-eclampsia, Caesarean section, and birth injury, while postpartum complications to offspring include macrosomia, shoulder dystocia, hyperinsulinemia, hypoglycemia, and hyperbilirubinemia [7,8,9]. In the long term, both mothers and their offspring are predisposed to metabolic conditions such as obesity, type 2 diabetes (T2D), and cardiovascular disease [10]. Estimates are that approximately 30% of offspring [11] and more than 70% of women with previous cases of GDM [12] are predisposed to develop type T2D later in life, thus posing significant health and economic burdens to health systems.

Recently, several studies provided evidence that early detection and treatment of GDM improve health outcomes [13]. Consequently, universal screening for GDM is advocated by most international organizations [14]. However, only women who have traditional risk factors for GDM (obesity, ethnicity, advanced maternal age, glycosuria, and previous adverse pregnancy outcomes) [15,16] are recommended for the OGTT in resource-limited settings. Unfortunately, these risk factors have poor predictive value, resulting in many women with GDM not receiving appropriate treatment [17]. Thus, there is significant impetus to identify biomarkers of GDM. Serum proteins such as adiponectin, insulin, sex hormone globulin, C-reactive protein, and glycosylated fibronectin are widely studied [15,18,19,20], while the diagnostic utility of glycated hemoglobin (HbA1c) was also explored [21]. However, these potential biomarkers are yet to achieve clinical applicability. Evidence for genetic susceptibility [22] and dysregulated epigenetic regulation, in particular DNA methylation [23] and miRNAs [24], is increasingly being reported during GDM, sparking interest in their use as molecular biomarkers.

## 3. Characteristics of Ideal Biomarkers

Biomarkers are indicators of normal biological processes that can be used to detect disease or other biological states of organisms. They are considered clinically useful because they can potentially predict or diagnose disease, give insight into the pathophysiology of disease, and can be used to monitor pharmacological responses to therapeutic intervention or to predict clinical outcome [25]. Recent advancements in molecular biology led to the development of molecular biomarkers that are easily measured in biological fluids such as whole blood, plasma, and serum. The ideal biomarker should be cost effective and reproducible, easily accessible through non-invasive methods, stably expressed in biological fluids, sensitive to relevant changes in disease state, provide early detection of disease before clinical symptoms arise, and have the ability to differentiate between disease pathologies [26,27]. Commercial kits for SNPs [28], DNA methylation [29], and miRNAs [30] are already clinically available for a number of other disorders.

## 4. Single-Nucleotide Polymorphisms

Single-nucleotide polymorphisms (SNPs) refer to alterations in the DNA sequence at individual nucleotide bases. They are the most common genetic variation, with over 10 million SNPs present in the human genome [31]. In most cases SNPs are silent, not altering the function or expression of genes [32], while others are biologically functional, and can lead to altered protein function and disease. The search for SNPs that influence disease susceptibility and outcome is a field of active research. Several studies provided evidence that SNPs are associated with metabolic conditions including obesity, T2D, and cardiovascular disease [33]. Variants in more than 50 and 80 loci were found to be associated with obesity [34] and T2D [35], respectively, and occur in genes that regulate glucose homeostasis and insulin signaling. 

### 4.1. Single-Nucleotide Polymorphisms and Gestational Diabetes Mellitus

Genetic variants are increasingly being implicated in the pathogenesis of GDM [4]. Evidence suggests that genetic alterations in genes responsible for metabolic changes during pregnancy predispose one to GDM. In this review, a total of 76 studies were identified that investigated SNPs during GDM, using the search terms previously stated. However, to increase the likelihood of reporting a true association, only SNPs investigated in two or more populations were reported. Thirty-four SNPs investigated in 49 studies met the inclusion criteria and are summarized in Table 1.

Genetic studies of the transcription factor 7 like 2 (*TCF7L2*) gene, which is arguably one of the most important T2D susceptibility genes [36], produced varying results in GDM [37,38,39,40,41,42,43,44,45,46]. *TCF7L2* encodes a transcription factor, which is involved in Wnt signaling, an important pathway that regulates glucose homeostasis. Twenty studies conducted in diverse populations screened four SNPs (rs7903146, rs4506565, rs7901695, and rs12255372) in the *TCF7L2* gene. Four of the eight studies that investigated rs7903146 showed an association between the T allele and GDM [37,38,43,44]. The other studies failed to observe an association between rs7903146 and GDM, possibly due to small sample size and a lack of statistical power. [40,41,42]. Both studies investigating rs4506565 reported an association between the T allele and GDM [37,41]. One of the five studies investigating rs7901695 found an association between GDM and the T allele in American Caucasians [46], while one study found that the C allele, rather than the T allele, was associated with GDM in a large Swedish population [43]. The three studies that did not show an association had relatively small sample sizes [40,41,45]. Of the five studies investigating rs12255372, two showed an association between the T allele and GDM, one was conducted in a large Swedish population and the other in a small Mexican population [42,43]. However, these results were not replicated in studies conducted in Russian, Spanish, or Brazilian populations [39,41,47] of moderate size, suggesting that ethnic or other confounding factors underlie these differences. The T allele is associated with decreased insulin production and altered hepatic gluconeogenesis [48], and therefore, is a good candidate for further research in larger cohorts, despite these conflicting results obtained in these studies. 

Adiponectin *(ADIPOQ*) is an adipokine that regulates glucose and lipid metabolism [49,50], which was associated with GDM in many studies. Three SNPs within the *ADIPOQ* gene, rs1501299, rs266729, and rs2241766, were investigated in eight studies. Markedly, all six studies that investigated rs266729 [51,52] and rs2241766 [52,53,54,55] found that the G allele was associated with GDM in various populations. Both studies investigating rs1501299 showed no association between this SNP and GDM [51,52].

The melanotonin receptor 1B gene (*MTNR1B*) encodes one of the receptors for melatonin, a hormone that is involved in regulating circadian rhythms, insulin signaling, and glucose metabolism, amongst others [56]. Two SNPs, rs10830963 and rs1387153, within the *MTNR1B* gene were investigated. Eight of the nine studies that screened rs10830963 showed that the G allele was associated with an increased risk for GDM in several Caucasian populations [37,39,44,46,57,58], as well as in Chinese and South Korean populations [59,60]. However, Wang et al. found that this SNP was not associated with GDM in a different Chinese population [61]. The three studies that investigated rs1387153 reported an association between the T allele and GDM [37,39,60]. Variants in *MTNR1B*, particularly the G allele of rs10830963, were previously shown to be associated with increased fasting glucose concentrations and reduced beta-cell function in Caucasians [62]. 

Glucokinase *(GCK*) and the glucokinase regulator *(GCKR*) play critical roles in glucose processing in the liver [63]. Two variants, rs1799884 and rs4607517, within the *GCK* gene were studied for GDM. For rs1799884, the minor allele, reported as either T [39] or A [64], was associated with an increased risk of GDM. Tarnowski et al. also showed a trend toward a significant association between the T allele and risk of GDM in a Polish population [65]. However, a large study in a Finnish population showed no association between rs1799884 and GDM [44]. No association between rs4607517 and GDM was observed [44,61]. Within the *GCKR* gene, the C allele of rs780094 was associated with an increased risk of GDM in Malaysian, American Caucasian, and Brazilian populations [46,66,67], but not in studies conducted in Polish or Finnish populations [44,65]. The C allele was increased in women with GDM from the Polish population, but this did not reach significance due to a lack of statistical power. 

The association between genetic variants within the fat mass and obesity-associated (*FTO*) gene and metabolic syndrome is widely reported [68]. *FTO* encodes an alpha-ketoglutarate-dependent dioxygenase, which plays a role in adipocyte development and function [69]. Three SNPs within the *FTO* gene were studied for GDM. Of the six studies investigating rs9939609, one study in a Finnish population found an association between the A allele and an increased risk for GDM [44], another study in a small Spanish population found an association between the T allele and GDM [41], while four studies reported no association [38,39,47,70]. Discrepancies between the studies are possibly due to ethnic and genotyping method differences. None of the studies investigating rs8050136 and rs1421085 found an association between these SNPs and GDM [45,47,70].

Insulin receptor substrate 1 (IRS1) is a protein that plays a key role in transmitting signals from the insulin and insulin-like growth factor-1 receptors to intracellular pathways that are associated with insulin response and risk of T2D [71]. Two genetic variants, rs1801278 and rs7578326, within *IRS1* were investigated during GDM. For rs1801278, the T allele was associated with an increased risk of GDM [72] in a Saudi Arabian population, but not in a Russian population [39], while, for rs7578326, the G allele was associated with a decreased risk of GDM in an Austro-Hungarian population [58], but not in a Finnish population [44]. As previously stated, these conflicting results may be due to population and genotyping method differences. 

Potassium voltage-gated channel subfamily Q member 1 (*KCNQ1*) plays a role in insulin secretion, and variants of *KCNQ1* are associated with decreased insulin secretion and increased susceptibility to T2D [73]. Two variants, rs2237895 and rs2237892, were investigated in different populations in four studies. In both variants, the C allele was associated with an increased risk of GDM [74,75,76]. The solute carrier family 30 member 8 (*SLC30A8*) gene encodes a zinc transporter protein that plays a role in insulin secretion, and variants of the gene are associated with T2D risk [77]. Rs13266634 was investigated in three studies with varying results. One study showed that the T allele was associated with a decreased risk of GDM in an Austro-Hungarian population, while the C allele was found to be associated with an increased risk of GDM in a large Swedish population [58,78]. A large Finnish population showed no association between rs13266634 and GDM [44].

As illustrated in Table 1, SNPs in 13 other genes were investigated in two studies; however, these showed either a positive association in one study only, or no association with GDM. Of these, SNPs within nine genes, cyclin-dependent kinase 5 *(CDK5*) regulatory-subunit-associated protein 1 like (*CDKAL1*), calpain 10 (*CAPN10*), potassium voltage-gated channel subfamily J member 11 (*KCNJ11*), retinol-binding protein 4 (*RBP4*), group-specific component (*GC*), serine/threonine kinase 11 (*STK11*), macrophage migration inhibitory factor (*MIF*), CDK inhibitor 2A/2B (*CDKN2A/2B*), and insulin-like growth factor 2 messenger RNA (mRNA)-binding protein 2 (*IGF2BP2*), were associated with GDM in one population only, while SNPs within four genes, cluster of differentiation 36 *(CD36*) molecule , peroxisome proliferator-activated receptor gamma 2 (*PPARG2*), vitamin D receptor (*VDR*), cell division cycle 123 homolog/calmodulin-dependent protein kinase ID (*CDC123*/*CAMK1D*), were not associated with GDM in any of the populations investigated.

### 4.2. Limitations of Single-Nucleotide Polymorphisms

There are inherent limitations in genetic association studies, particularly in studies of polygenic and multifactorial diseases such as GDM. As stated above, these limitations include inadequate sample size to detect statistically significant associations, and differences in allele frequencies and disease etiology between ethnicities, which may explain why many genetic associations are not reproducible across populations. Furthermore, GDM diagnosis is not standardized internationally; thus, different diagnostic criteria could have contributed to the discordant results observed between studies. Importantly, genetic variants do not solely contribute to the development of complex diseases, and it is widely believed that disease arise due to the interaction of genetic predisposition and environmental factors [79]. Thus, to accurately assess risk of GDM, biological and environmental factors, such as maternal age and diet [39], should be considered together with genetic variants.

Despite the variable results obtained across studies, many of the variants found to be associated with GDM, are also associated with T2D, supporting their biological plausibility. Therefore, while the etiology of GDM may differ from T2D, the genetic pathways through which the symptoms manifest are likely to overlap. In this review, only studies that profiled SNPs in DNA extracted from whole blood were reported on. However, the use of less invasive sources of genetic material, such as buccal swabs, is acknowledged [80]. Furthermore, this review only included SNPs reported in two or more studies, and may have overlooked other important SNPs possibly associated with GDM.

## 5. DNA Methylation

DNA methylation, the most widely studied and best characterized epigenetic mechanism, occurs via the addition of a methyl group to the fifth carbon position of a cytosine residue within cytosine–phosphate–guanine (CpG) dinucleotides [97]. The process is catalyzed by the enzyme DNA methyltransferase (DNMT), with S-adenosyl-methionine serving as the methyl donor. Methylation of CpG islands, which are regions with high levels of CpG dinucleotides primarily in the promoter regions of genes, is generally associated with transcriptional repression due to altered protein binding to target sites on DNA [98,99]. DNA methylation is a reversible process [100]. Ten-eleven translocation (TET) methylcytosine dioxygenases are able to cause the oxidation and demethylation of methylated cytosine to 5-hydroxymethylcytosine [100], which is associated with gene activation. Recently, DNA methylation of CpG-poor islands was identified downstream of active promoters, either within (intragenic) or between (intergenic) genes, although the role of methylation in these regions are not fully elucidated [101]. Approximately 55–90% of all CpG dinucleotides within CpG islands are methylated, constituting about 3% of the genome. Global DNA hypomethylation is associated with genomic and chromosomal instability, while DNA methylation within the promoters of genes is generally associated with gene silencing. Both aberrant global and gene-specific DNA methylation was shown to be associated with metabolic conditions such as obesity [102], T2D [103], and cardiovascular disease [104]. Thus, characterization of altered DNA methylation during disease processes could give insight into the pathophysiology of disease, and reveal novel diagnostic, prognostic, and therapeutic targets. 

### 5.1. DNA Methylation and Gestational Diabetes Mellitus

DNA methylation during pregnancy plays a key role in modulating the transcriptional potential of the genome, and is known to affect gene expression pathways associated with a range of pathophysiological processes such as GDM [49,105]. Several studies demonstrated that DNA methylation is altered in the placenta and cord blood of women with GDM compared to women with normoglycemic pregnancies [23,106,107,108,109]. Intrauterine exposure to GDM leads to long-lasting effects in the offspring and increases risk of disease in later life, possibly mediated by DNA methylation [110,111]. Importantly, it was demonstrated that physiological and DNA methylation changes that occur during pregnancy are reflected in whole blood [112], thus increasing interest in screening maternal blood for biomarkers of GDM. DNA methylation profiling in pregnancies complicated by GDM is a relatively new research field, with limited studies conducted in maternal whole blood. Studies that investigated DNA methylation in whole blood of women with GDM are summarized in Table 2.

Global DNA methylation provides an estimate of overall genomic methylation and is relatively easy and cost-effective to measure [113]. Currently, the only study that investigated global DNA methylation during GDM was conducted in our laboratory [114]. The study showed that global DNA methylation was not associated with GDM in a South African population, suggesting that the method may be too crude to detect subtle glucose intolerance, and that gene-specific methylation is warranted in this population. Genome-wide DNA methylation profiling in maternal blood during GDM was conducted using methylation bead chip arrays [115,116,117]. Methylation bead chip arrays can interrogate between 27,000 and 850,000 CpG sites across the genome at a single-nucleotide resolution. In one of the earliest studies using bead chip arrays, Enquobahrie et al. reported that DNA methylation changes occurred early during pregnancy in six women with repeat pregnancies, one of which was complicated by GDM [116]. They reported that 17 CpG sites were hypomethylated and 10 CpG sites were hypermethylated between GDM and normal pregnancies within the same women. Novel genes related to these CpG sites were found to be associated with cell cycle, cell morphology, cell assembly, cell organization, and cell compromise. Subsequently, using a newer bead chip array containing more CpG sites, Kang et al. showed that 200 CpGs corresponding to 151 genes were differentially methylated in women with GDM (*n* = 8) compared to controls (*n* = 8). Amongst the differentially methylated genes were interleukin-6 (*IL-6*) and interleukin-10 (*IL-10*), which are key pro-inflammatory and anti-inflammatory cytokines, respectively [115]. These cytokines function in a wide variety of inflammatory-associated diseases, including obesity and T2D. Moreover, a different study by Kang et al. showed that decreased methylation of *IL-10* during GDM was associated with increased serum *IL-10* concentrations at the end of pregnancy [118]. *IL-10* serum concentrations were shown to vary during pregnancy, suggesting that this cytokine plays an important role in the development of GDM. In another study using bead chip arrays, 100 differentially methylated CpG sites corresponding to 66 genes were identified in women with GDM (*n* = 11) compared to controls (*n* = 11) [117]. Using more stringent statistical criteria to prioritize methylation sites, a total of five CpG sites within the constitutive photomorphogenic homolog subunit 8 (*COPS8*), phosphoinositide 3-kinase regulatory subunit 5 (*PIK3R5*), 3-hydroxyanthranilate 3,4-dioxygenase (*HAAO*), coiled-coil domain containing 124 (*CCDC124*), and chromosome 5 open reading frame 34 (*C5orf34*) genes were identified and validated using pyrosequencing. Since blood for DNA methylation profiling was collected prior to GDM diagnosis, these CpG sites may prove useful as predictive biomarkers for GDM. However, their candidacy as biomarkers requires validation in larger studies.

### 5.2. Limitations of DNA Methylation

Although studies show that DNA methylation has potential as a diagnostic and prognostic biomarker, they are not without limitations [119]. Several factors, including small sample size, lack of validation, differences in ethnicity, method of quantification, and timing of methylation analysis during pregnancy, hinder reproducibility of findings across studies. Another limitation of the studies included in this review is the use of whole blood, which consists of a mixture of cell types such as lymphocytes, erythrocytes, and platelets, and may confound methylation analysis [120]. Thus, future studies should consider purification of blood-cell populations to separate specific cell types. Currently, there is no consensus on the best method to use for DNA methylation analysis. While global DNA methylation can easily be measured using crude DNA preparations, it is a measure of overall genomic methylation and does not offer the resolution required to detect subtle DNA methylation differences within genes [121]. In contrast, locus-specific DNA methylation methods such as bead chip arrays and pyrosequencing are expensive, requiring sophisticated equipment and bioinformatics expertise.

## 6. MicroRNAs

MiRNAs are short, highly conserved non-coding RNA molecules, approximately 22 nucleotides in length, which are powerful mediators of biological function. They regulate gene expression through post-transcriptional mechanisms by binding to the 3′ untranslated region (UTR) of messenger RNA (mRNA), inducing gene silencing through translational repression or mRNA degradation [122]. This interaction is dependent on the complementarity of the miRNA to the “miRNA seed region”, a region of seven or eight nucleotides contained within the 3′ UTR of mRNA. MiRNA binding requires a number of nucleotides to match the sequence flanking the seed region to direct the specificity of miRNA–mRNA interactions [123,124]. Since their initial discovery in *Caenorhabditis elegans* in 1993 [125], over 2000 miRNAs were identified in humans, and they are believed to regulate about one-third of the genome [126]. 

MiRNAs are master regulators that control many biological processes including cell proliferation, differentiation, apoptosis, and development [127]. Moreover, they regulate genes involved in metabolic processes such as glucose homeostasis, insulin signaling, pancreatic beta-cell function, lipid metabolism, and inflammation [128]. Their dysregulation was reported during many metabolic conditions, including obesity, T2D, and cardiovascular disease [129,130,131]. Although they exert their function intracellularly, several studies identified extracellular circulating miRNAs, which sparked interest in their use as biomarkers of disease [132]. Circulating miRNAs are associated with various complexes such as lipoproteins, exosomes, apoptotic bodies, microvesicles, and ribonucleoproteins such as Argonaute (Ago)1–4 or nucleophosphin 1 (NPM1), which serve to protect these miRNAs from nuclease degradation, and act as carriers to transport them to their target mRNAs. This suggests that miRNAs function in cell-to-cell communication, regulating gene expression in neighboring cells by either acting locally (paracrine or autocrine signaling) or at a distance (endocrine/exocrine) [132,133].

### 6.1. MicroRNAs and Gestational Diabetes Mellitus 

MiRNAs are important metabolic and developmental regulators during pregnancy, and were shown to play a role in the development of GDM. In 2013, genome-wide analysis demonstrated that more than 600 miRNAs are expressed in the placenta [134]. Recently, Poirer et al. reviewed placental miRNAs that are dysregulated during pregnancy and GDM [135]. The placenta plays an important role in maternal metabolic adaptation to pregnancy, and differential expression of placental miRNAs are believed to partly underlie these physiological changes. Placental miRNAs are released into maternal circulation [112]; thus, these miRNAs hold potential as biomarkers of placental dysfunction and GDM. Studies reporting circulating miRNA expression during GDM are summarized in Table 3.

In 2011, Zhao et al. were the first to profile the expression of serum miRNAs during GDM [136]. Using Taqman low-density arrays, followed by confirmation with individual qRT-PCR, they identified three miRNAs, miR-132, miR-29a, and miR-222, that were significantly downregulated in Chinese women with GDM (*n* = 24) compared to controls (*n* = 24) [136]. The differential expressions of miR-29a and miR-222 were validated in an internal and two external validation cohorts. These miRNAs are thought to play a role in glucose homeostasis, insulin sensitivity, and beta-cell function [136]. A number of studies in other populations replicated these experiments with conflicting results. Recently, Pheiffer et al. reported decreased expression of miR-132, miR-29a, and miR-222 in the serum of South African women with GDM (*n* = 28) compared to controls (*n* = 53); however, only the latter was statistically significant [5]. These findings demonstrate that the expression of these serum miRNAs are shared across South African and Chinese populations. In contrast to Zhao et al., Tagnoma et al. showed that miR-222 expression was increased in plasma of women with GDM (*n* = 13) compared to controls (*n* = 9) [137]. Wander et al. observed no differences in the expression of miR-222 or miR-29a in the plasma of American Caucasian women with GDM (*n* = 36) compared to controls (*n* = 80) [138]. These discrepancies may be due to differences in biological samples used (serum or plasma), gestational age, or other unknown factors not accounted for. 

Zhu et al. used high-throughput sequencing and qRT-PCR to investigate miRNAs in pooled plasma samples of Chinese women with (*n* = 10) or without (*n* = 10) GDM between 16 and 19 weeks of gestation. Five miRNAs (miR-16, miR-17, miR-19a, miR-19b, and miR-20a) were significantly upregulated in GDM compared to controls [139]. Bioinformatic analysis revealed that the targets of these miRNAs are associated with mitogen-activated protein kinase (MAPK), insulin, transforming growth factor beta (TGF-β), and mammalian target of rapamycin (mTOR) signaling pathways, providing insight into the role of these miRNAs in GDM. Cao et al. investigated miR-16, miR-17, and miR-20a in a larger cohort of Chinese women at 16–19 weeks, 20–24 weeks, and 24–28 week of gestation and found sustained increased expression in the plasma of women with GDM (*n* = 85) compared to controls (*n* = 72) at all the measured time points. However, they did not observe differences in the expressions of miR-19a and miR-19b [140], as previously reported by Zhu et al. More recently, Pheiffer et al. reported conflicting results. The expression of all five miRNAs were decreased in South African women with GDM; however, only the decreased expression of miR-20a was statistically significant [5]. 

Functional analyses of these miRNAs provided support for their role in the development of GDM [141,142,143,144]. Many other miRNAs were reported to exhibit altered expression during GDM, although these were identified in single studies only (Table 3). 

### 6.2. Limitations of Circulating microRNA Profiling

The studies reviewed above highlight several miRNA candidates as biomarkers for GDM. However, the results are often discordant, possibly due to the different sample types and sizes, gestational age, and the methods of analysis used. 

Differences in miRNA expression were reported in serum and plasma, suggesting that factors during the coagulation process could influence expression [145]. Currently, there is no consensus on the best quantification method to use when profiling circulating miRNAs. Different methods of quantification are known to vary in sensitivity and specificity [146], which may impact the accuracy and interpretation of the data. Moreover, data normalization presents a significant challenge for the analysis of circulating miRNA profiling. Although strategies using exogenous miRNAs such as *C. elegans* miR-39 were proven to be less variable than endogenous reference genes, no ideal normalization strategy exists [147]. Thus, standardized guidelines for miRNA profiling would aid in the biological interpretation of miRNA data.

## 7. Current Perspectives and Future Recommendations

Advances in molecular biology resulted in the identification of several molecular biomarkers for disease. Of these genetic variants, DNA methylation and miRNAs are widely studied during GDM [148,149,150]. These molecular markers are stably expressed in biological fluids and hold potential as diagnostic or prognostic biomarkers of GDM. As reviewed above, many studies provided evidence to support the use of these markers as biomarkers of GDM. However, despite these favorable results, molecular biomarkers face many challenges, which hinder their candidacy as biomarkers, and that must be addressed before they can be used clinically. As outlined above, SNPs, DNA methylation, and miRNAs are all impacted by ethnicity and environmental factors. Furthermore, technical challenges during analysis contribute to inaccurate data and lack of reproducibility. Thus, standardization of analytical methods is critical when profiling molecular biomarkers. Moreover, large prospective cohort studies, conducted in populations with different ethnicities and environmental factors, are warranted to identify robust markers that are not influenced by these factors. The ideal biomarker for GDM would most likely be a combination of several molecular biomarkers to overcome the lack of sensitivity and specificity of individual factors. For example, a single miRNA regulates up to 200 different genes [151]; thus, miRNAs found to be associated with GDM are non-specific and may possibly be involved in other conditions as well. To increase the predictive power of molecular biomarkers, future studies should consider using a combination of these markers in risk stratification models for predicting GDM risk.

## 8. Conclusions

GDM is a growing public health problem worldwide. The short- and long-term consequences of GDM are likely to have an immediate negative impact on health systems, and, in addition, present a major reservoir of future disease. Screening and treatment of GDM leads to improved pregnancy outcomes [13]; thus, universal screening is widely advocated as a strategy to prevent adverse consequences. A growing body of evidence supports the use of SNPs, DNA methylation, and miRNAs as biomarkers that could aid in the early detection of GDM, thus facilitating intervention strategies to better manage GDM and improve health outcomes. Despite their potential, these molecular biomarkers face several challenges that need to be addressed before they can become clinically applicable. However, rapid technological advances could overcome these challenges and lead to the development of a quick, cost-effective point-of-care test that could accurately identify women at high risk for GDM during early pregnancy. The establishment of an international body to standardize analytical conditions for molecular biomarkers, and large prospective cohort studies in different populations are required.

## Figures and Tables

**Table 1:** Studies reporting on single-nucleotide polymorphisms (SNPs) profiled in two or more populations with gestational diabetes mellitus (GDM).

Author	Gene	SNP Identification	Country	Detection Method	Case/Control	Associated Allele or Genotype	Risk for GDM
Ding et al., 2018 [37]	*TCF7L2*	rs7903146	Denmark and USA	qRT-PCR	2636/6086	T allele	Increased
Franzago et al., 2018 [38]			Italy	HRM	104/124	T allele	Increased
Popova et al., 2017 [39]			Russia	qRT-PCR	278/179	No association	-
Michalak-Wojnowska et al., 2016 [40]			Poland	qRT-PCR	50/26	No association	-
Pagán et al., 2014 [41]			Spain	Sequencing	45/25	No association	-
Reyes-López et al., 2014 [42]			Mexico	RFLP	90/108	No association	-
Papadopoulou et al., 2011 [43]			Sweden	qRT-PCR	826/1185	T allele	Increased
Huopio et al., 2013 [44]			Finland	MassARRAY	533/407	T allele	Increased
Ding et al., 2018 [37]		rs4506565	Denmark and USA	qRT-PCR	2636/6086	T allele	Increased
Pagán et al., 2014 [41]			Spain	Sequencing	45/25	T allele	Increased
Anghebem-Oliveira, et al., 2017 [45]		rs7901695	Brazil	qRT-PCR	127/125	No association	-
Michalak-Wojnowska et al., 2016 [40]			Poland	qRT-PCR	50/26	No association	-
Pagán et al., 2014 [41]			Spain	Sequencing	45/25	No association	-
Stuebe et al., 2014 [46]			USA African American (AA) and Caucasian (C)	MassARRAY	26/362 (AA) and 56/843 (C)	No association (AA) T allele (C)	- Increased
Papadopoulou et al., 2011 [43]			Sweden	qRT-PCR	805/1116	C allele	Increased
Popova et al., 2017 [39]		rs12255372	Russia	qRT-PCR	278/179	No association	-
de Melo et al., 2015 [47]			Brazil	qRT-PCR	200/200	No association	-
Pagán et al., 2014 [41]			Spain	Sequencing	45/25	No association	-
Reyes-López et al., 2014 [42]			Mexico	RFLP	90/108	T allele	Increased
Papadopoulou et al., 2011 [43]			Sweden	qRT-PCR	826/1185	T allele	Increased
Pawlik et al., 2017 [51]	*ADIPOQ*	rs1501299	Poland	qRT-PCR	204/207	No association	-
Beltcheva et al., 2014 [52]			Bulgaria	qRT-PCR	130/130	No association	-
Pawlik et al., 2017 [51]		rs266729	Poland	qRT-PCR	204/207	G allele	Increased
Beltcheva et al., 2014 [52]			Bulgaria	qRT-PCR	130/130	G allele	Increased
Takshid et al., 2015 [53]		rs2241766	Iran	RFLP	65/70	G allele	Increased
Han et al., 2014 [55]			China	RFLP	128/140	G allele	Increased
Beltcheva et al., 2014 [52]			Bulgaria	qRT-PCR	130/130	G allele	Increased
Low et al., 2011 [54]			Malaysia	RFLP	26/53	G allele	Increased
Ding et al., 2018 [37]	*MTNR1B*	rs10830963	Denmark and USA	qRT-PCR	2636/6086	G allele	Increased
Li et al., 2018 [59]			China	Sequencing	215/243	G allele	Increased
Tarnowski et al., 2017 [57]			Poland	qRT-PCR	204/207	G allele	Increased
Rosta et al., 2017 [58]			Hungary and Austria	KASP	287/533	G allele	Increased
Popova et al., 2017 [39]			Russia	qRT-PCR	278/179	G allele	Increased
Stuebe et al., 2014 [46]			USA African American (AA) and Caucasian (C)	MassARRAY	26/362 (AA) and 56/843 (C)	No association (AA) G allele (C)	- Increased
Wang et al., 2011 [61]			China	qRT-PCR	725/1039	No association	-
Kim et al., 2011 [60]			South Korea	qRT-PCR	928/990	G allele	Increased
Huopio et al., 2013 [44]			Finland	MassARRAY	533/407	G allele	Increased
Ding et al., 2018 [37]		rs1387153	Denmark and USA	qRT-PCR	2636/6086	T allele	Increased
Popova et al., 2017 [39]			Russia	qRT-PCR	278/179	T allele	Increased
Kim et al., 2011 [60]			South Korea	qRT-PCR	928/990	T allele	Increased
Tarnowski et al., 2017 [65]	*GCK*	rs1799884	Poland	qRT-PCR	204/207	No association	-
Popova et al., 2017 [39]			Russia	qRT-PCR	278/179	T allele	Increased
Han et al., 2015 [64]			China	PCR Invader assay	948/975	A * allele	Increased
Huopio et al., 2013 [44]			Finland	MassARRAY	533/407	No association	-
Wang et al., 2011 [61]		rs4607517	China	qRT-PCR	725/1039	No association	-
Huopio et al., 2013 [44]			Finland	MassARRAY	533/407	No association	-
Jamalpour et al., 2018 [66]	*GCKR*	rs780094	Malaysia	MassARRAY	267/855	C allele	Increased
Tarnowski et al., 2017 [65]			Poland	qRT-PCR	204/207	No association	-
Anghebem-Oliveira et al., 2017 [67]			Brazil	qRT-PCR	127/125	C allele	Increased
Stuebe et al., 2014 [46]			USA African American (AA) and Caucasian (C)	MassARRAY	26/362 (AA) and 56/843 (C)	No association C allele	- Increased
Huopio et al., 2013 [44]			Finland	MassARRAY	533/407	No association	-
Franzago et al., 2018 [38]	*FTO*	rs9939609	Italy	HRM	104/124	No association	-
Saucedo et al., 2017 [70]			Mexico	qRT-PCR	80/80	No association	-
Popova et al., 2017 [39]			Russia	qRT-PCR	278/179	No association	-
de Melo et al., 2015 [47]			Brazil	qRT-PCR	200/200	No association	-
Pagán et al., 2015 [41]			Spain	Sequencing	45/25	T allele	Increased
Huopio et al., 2013 [44]			Finland	MassARRAY	533/407	A allele	Increased
Saucedo et al., 2017 [70]		rs8050136	Mexico	qRT-PCR	80/80	No association	-
de Melo et al., 2015 [47]			Brazil	qRT-PCR	200/200	No association	-
Saucedo et al., 2017 [70]		rs1421085	Mexico	qRT-PCR	80/80	No association	-
Anghebem-Oliveira et al., 2017 [45]			Brazil	qRT-PCR	127/125	No association	-
Popova et al., 2017 [39]	*IRS1*	rs1801278	Russia	qRT-PCR	278/179	No association	-
Alharbi et al., 2014 [72]			Saudi Arabia	RFLP	200/300	T allele	Increased
Huopio et al., 2013 [44]		rs7578326	Finland	MassARRAY	533/407	No association	-
Rosta et al., 2017 [58]			Hungary and Austria	KASP	287/533	G allele	Decreased
Fatima et al., 2016 [74]	*KCNQ1*	rs2237895	Pakistan	RFLP/sequencing	208/429	C allele	Increased
Kwak et al., 2010 [75]			South Korea	qRT-PCR	869/632	No association	-
Ao et al., 2015 [76]		rs2237892	China	MassARRAY	562/453	C allele	Increased
Kwak et al., 2010 [75]			South Korea	qRT-PCR	869/632	C allele	Increased
Rosta et al., 2017 [58]	*SLC30A8*	rs13266634	Hungary and Austria	KASP	287/533	T allele	Decreased
Dereke et al., 2016 [78]			Sweden	RFLP	776/511	C allele	Increased
Huopio et al., 2013 [44]			Finland	MassARRAY	533/407	No association	-
Noury et al., 2018 [81]	*CDKAL1*	rs7754840	Egypt	qRT-PCR	47/51	No association	-
Rosta et al., 2017 [58]			Hungary and Austria	KASP	287/533	C allele	Increased
Popova et al., 2017 [39]			Russia	qRT-PCR	278/179	No association	-
Wang et al., 2011 [61]			China	qRT-PCR	725/1039	No association	-
Huopio et al., 2013 [44]			Finland	MassARRAY	533/407	No association	-
Castro-Martinez et al., 2018 [82]	*CAPN10*	SNP43	Mexico	qRT-PCR & RFLP	116/83	No association	-
Leipold et al., 2004 [83]			Austria	RFLP	100/100	No association	-
Castro-Martinez et al., 2018 [82]		SNP63	Mexico	qRT-PCR & RFLP	116/83	No association	-
Leipold et al., 2004 [83]			Austria	RFLP	40/40	C allele	Increased
Huopio et al., 2013 [44]			Finland	MassARRAY	533/407	No Association	-
Lenin et al., 2018 [84]	*KCNJ11*	rs5219	India	RFLP	230/240	T allele	Increased
Popova et al., 2017 [39]			Russia	qRT-PCR	278/179	No association	-
Huopio et al., 2013 [44]			Finland	MassARRAY	533/407	No association	-
Saucedo et al., 2014 [85]	*RBP4*	rs3758539	Mexico	qRT-PCR	100/100	No association	-
Ping et al., 2012 [86]			China	LDR	505/687	G allele	Increased
Hiraoka et al., 2011 [87]			USA Caucasian (C), Filipino (F), and Pacific Islander (PI)	qRT-PCR	88/315 (C), 82/286 (F), and 19/32 (PI)	No association	-
Shi et al., 2016 [88]	*GC*	rs16847024	China	MassARRAY	964/1021	T allele	Increased
Wang et al., 2015 [89]			China	qRT-PCR	692/802	No association	-
Alharbi et al., 2015 [90]	*STK11*	rs8111699	Saudi Arabia	RFLP	200/300	No association	-
Bassols et al., 2013 [91]			Spain	qRT-PCR	243/318	G allele	Decreased
Aslani et al., 2011 [92]	*MIF*	rs1007888	Iran	PCR-SSP	147/169	G allele	Increased
Huopio et al., 2013 [44]			Finland	MassARRAY	533/407	No association	-
Noury et al., 2018 [81]	*CDKN2A/2B*	rs10811661	Egypt	qRT-PCR	47/51	No association	-
Ye et al., 2016 [93]			Poland	qRT-PCR	204/207	C allele	Decreased
Huopio et al., 2013 [44]			Finland	MassARRAY	533/407	No association	-
Popova et al., 2017 [39]	*IGF2BP2*	rs4402960	Russia	qRT-PCR	278/179	No association	-
Wang et al., 2015 [89]			China	qRT-PCR	725/1039	T allele	Increased
Huopio et al., 2013 [44]			Finland	MassARRAY	533/407	No association	-
Bartákova et al., 2018 [94]	*CD36*	rs1527479	Czech Republic	qRT-PCR	293/70	No association	-
Yang et al., 2018 [95]			China	qRT-PCR	209/215	No association	-
Franzago et al., 2018 [38]	*PPARG2*	rs1801282	Italy	HRM	104/124	No association	-
Anghebem-Oliveira et al., 2017 [45]			Brazil	qRT-PCR	127/125	No association	-
Shi et al., 2016 [88]	*VDR*	rs739837	China	MassARRAY	964/1021	No association	-
Wang et al., 2015 [89]			China	qRT-PCR	692/802	No association	-
Tarnowski et al., 2017 [96]	*CDC123/CAMK1D*	rs1277970	Poland	qRT-PCR	204/207	No association	-
Huopio et al., 2013 [44]			Finland	MassARRAY	533/407	No association	-

RFLP—restriction fragment length polymorphism of PCR-amplified fragments; KASP—kompetitive allele specific PCR; qRT-PCR—quantitative real-time PCR (TaqMan allelic discrimination assay); LDR—ligase detection reaction; HRM—high-resolution melt-curve analysis; MassARRAY—Sequenom MassARRAY iPLEX platform; USA—United States of America; PCR invader assay—invasive cleavage reaction which uses a structure-specific flap endonuclease. * A is the minor allele also reported as T. *TCF7L2*—transcription factor 7 like 2; *ADIPOQ*—adiponectin; *MTNR1B*—melatonin receptor 1B; *CAPN10*—calpain 10; *CDKAL1*—cyclin-dependent kinase 5 (CDK5) regulatory-subunit-associated protein 1 like; *CDKN2A/2B*—CDK inhibitor 2A/2B; *FTO*—fat mass And obesity-associated; *GC*—group-specific component (vitamin-D-binding protein); *GCK*—glucokinase; *GCKR*—glucokinase Regulator; *IGF2BP2*—insulin-like growth factor 2 messenger RNA (mRNA)-binding protein 2; *IRS1*—insulin receptor substrate 1; *KCNJ11*—potassium voltage-gated channel subfamily J member 11; *KCNQ1*—potassium voltage-gated channel subfamily Q member 1; *RBP4*—retinol-binding protein 4; *SLC30A8*—solute carrier family 30 member 8; *STK11*—serine/threonine kinase 11; *MIF*—macrophage migration inhibitory factor; *CD36*—cluster of differentiation 36 molecule; *PPARG2*—peroxisome proliferator-activated receptor gamma 2; *VDR*—vitamin D receptor; *CDC123/CAMK1D*—cell division cycle 123 homolog/calmodulin-dependent protein kinase ID.

**Table 2:** Studies investigating DNA methylation in whole blood during gestational diabetes mellitus.

Author	Study Design	Country	Detection Method	Main Finding
Dias et al., 2018 [114]	63 GDM and 138 controls (~26 weeks gestation)	South Africa	Global DNA methylation using MDQ1 Imprint DNA Quantification Kit *	No difference in global DNA methylation between women with or without GDM. Global DNA methylation was associated with obesity and serum adiponectin concentrations.
Enquobahrie et al., 2015 [116]	6 women with 2 consecutive pregnancies with and without GDM (<20 weeks gestation)	United States	Illumina HumanMethylation27 BeadChip	17 CpG sites were hypomethylated and 10 CpG sites were hypermethylated in relation to GDM status
Kang et al., 2017 [115]	8 GDM and 8 controls (end of pregnancy)	Taiwan	Illumina Infinium HumanMethylationEPIC BeadChip	200 differentially methylated CpGs corresponding to 151 genes identified in women with GDM compared to controls
Kang et al., 2018 [118]	8 GDM and 24 controls (end of pregnancy)	Taiwan	MethyLight qRT-PCR assay	Decreased methylation of *IL-10* during GDM, which was associated with increased serum *IL-10* concentrations
Wu et al., 2018 [117]	11 GDM and 11 controls (12–16 weeks gestation)	United Kingdom	Illumina HumanMethylation450 BeadChip (450K) array and bisulfite pyrosequencing	100 differentially methylated CpGs corresponding to 66 genes were identified. Differential DNA methylation at 5 CpGs were validated in 8 of the 11 GDM women

qRT-PCR—quantitative real-time PCR; CpG—cytosine–phosphate–guanine; *IL-10*—interleukin-10; GDM—gestational diabetes mellitus. * Sigma-Aldrich. St. Louis, USA.

**Table 3:** Studies investigating circulating microRNAs (miRNAs) during gestational diabetes mellitus.

Author	Study Design	Country	Biological Source	Detection Method	Upregulated	Downregulated	No Significant Change	Normalization Control
Zhao et al., 2011 [136]	24 GDM and 24 controls (16–19 weeks gestation); 36 GDM and 36 controls (internal validation); 16 GDM and 16 controls (external validation)	China	Serum	Taqman low-density array, qRT-PCR	-	miR-29a, miR-132, miR-222	-	Cel-miR-39 (exogenous control)
Pheiffer et al., 2018 [5]	28 GDM and 53 controls (13–31 weeks gestation)	South Africa	Serum	qRT-PCR	-	miR-20a, miR-222	miR-16, miR-17, miR-19a, miR-19b, miR-29a, miR-132	Cel-miR-39 (exogenous control)
Tagnoma et al., 2018 [137]	13 GDM and 9 controls (23–31 weeks gestation)	Estonia	Plasma	qRT-PCR	let-7e, let-7g, miR-100, miR-101, miR-146a, miR-8a, miR-195, miR-222, miR-23b, miR-30b, miR-30c, miR-30d, miR-342, miR-423, miR-92a	-	-	Cel-miR-39 (exogenous control)
Wander et al., 2017 [138]	36 GDM and 80 controls (7–23 weeks gestation)	USA	Plasma	qRT-PCR	miR-155, miR-21		miR-146b, miR-517, miR-222, miR-210, miR-518a, miR-29a, miR-223, miR-126	Cel-miR-39 (exogenous control) and miR-423 (endogenous control)
Zhu et al., 2015 [139]	10 GDM and 10 controls (16–19 weeks gestation)	China	Plasma	Ion Torrent sequencing, qRT-PCR	miR-16, miR-17, miR-19a, miR-19b, miR-20a	-	-	miR-221 (endogenous control)
Cao et al., 2017 [140]	85 GDM and 72 controls (16–20, 20–24, and 24–28 weeks gestation)	China	Plasma	qRT-PCR	miR-16, miR-17, miR-20a	-	miR-19a miR-19b	RNU6 (endogenous control)
Sebastiani et al., 2017 [141]	21 GDM and 10 controls (24–33 weeks gestation)	Italy	Plasma	qRT-PCR	miR-330	-	miR-548c	miR-374, miR-320 (endogenous control)
Stirm et al., 2018 [142]	30 GDM and 30 controls (24–32 weeks gestation)	Germany	Whole blood	qRT-PCR	miR-340	-	-	RNU6B (endogenous control)
He et al., 2017 [143]	20 GDM and 20 controls	China	Whole blood	qRT-PCR	-	miR-494	-	RNU6 (endogenous control)
Lamadrid-Romero et al., 2018 [144]	67 GDM and 74 controls (16–20, 20–24, and 24–28 weeks gestation)	Not reported	Serum	qRT-PCR	miR-183, miR-200b, miR-125b, miR-1290	-	-	Cel-miR-39 (exogenous control)

GDM—gestational diabetes mellitus; qRT-PCR—quantitative real-time PCR.

## Abbreviations

GDM	Gestational diabetes mellitus
DNA	Deoxyribonucleic acid
SNPs	Single-nucleotide polymorphisms
OGTT	Oral glucose tolerance test
T2D	Type 2 diabetes
BMI	Body mass index
HbA1c	Glycated hemoglobin
qRT-PCR	Quantitative real-time PCR
RFLP	Restriction fragment length polymorphism
KASP	Kompetitive allele specific PCR
LDR	Ligase detection reaction
HRM	High-resolution melt-curve analysis
*TCF7L2*	Transcription factor 7 like 2
*ADIPOQ*	Adiponectin
*MTNR1B*	Melatonin receptor 1b
*CAPN10*	Calpain 10
*CD36*	Cluster of differentiation 36 molecule
*CDKAL1*	Cyclin-dependent kinase 5 regulatory subunit associated protein 1 like 1
*CDKN2A/2B*	Cyclin-dependent kinase inhibitor 2a/2b
*FTO*	Fat mass and obesity-associated
*GC*	Group-specific component
*GCK*	Glucokinase
*GCKR*	Glucokinase regulator
*IGF2BP2*	Insulin-like growth factor 2 messenger RNA (mRNA)-binding protein 2
*IRS1*	Insulin receptor substrate 1
*KCNJ11*	Potassium voltage-gated channel subfamily J member 11
*KCNQ1*	Potassium voltage-gated channel subfamily Q member 1
*PPARG2*	Peroxisome proliferator-activated receptor gamma 2
*RBP4*	Retinol-binding protein 4
*SLC30A8*	Solute carrier family 30 member 8
*STK11*	Serine/threonine kinase 11
*VDR*	Vitamin D receptor
*CDC123*	Cell division cycle 123 homolog
*CAMK1D*	Calmodulin-dependent protein kinase 1D
*MIF*	Macrophage migration inhibitory factor
DNMT	DNA methyltransferase
TET	Ten-eleven translocation
*IL-10*	Interleukin-10
*IL-6*	Interleukin-6
*COPS8*	Constitutive photomorphogenic homolog subunit 8
*PIK3R5*	Phosphoinositide 3-kinase regulatory subunit 5
*HAAO*	3-hydroxyanthranilate 3,4-dioxygenase
*CCDC124*	Coiled-coil domain containing 124
*C5orf34*	Chromosome 5 open reading frame 34
UTR	Untranslated region
mRNA	Messenger RNA
NPM1	Nucleophosphin 1
MAPK	Mitogen-activated protein kinase
TGF-β	Transforming growth factor beta
mTOR	Mammalian target of rapamycin

## Appendix A

**Table A1:** Search terms, including gestational diabetes mellitus, blood, single-nucleotide polymorphism, DNA methylation, and microRNAs, and corresponding synonyms searched.

**Concept 1:**	**Synonyms to be searched**
Gestational diabetes mellitus	Gestational diabetes mellitus
	Hyperglycemia during pregnancy
	Diabetes of pregnancy
	Glucose intolerance during pregnancy
	Maternal hyperglycemia
	Maternal hyperglycaemia
	Diabetes during pregnancy
**Concept 2:**	**Synonyms to be searched**
microRNAs	MicroRNAs or miRNAs
	Circulating microRNAs or miRNAs
	Circulating miRNAs
	Cell free microRNAs or miRNAs
	Small non-coding RNAs
	Circulating biomarkers
**Concept 3:**	**Synonyms to be searched**
DNA methylation	Global DNA methylation
	Gene-specific DNA methylation
	Genome-wide DNA methylation
**Concept 4:**	**Synonyms to be searched**
SNP	Single-nucleotide polymorphisms
	SNP genotyping
	Genetic DNA variation
	Genetic variants
**Concept 5:**	**Synonyms to be searched**
Biological markers	Whole blood
	Peripheral blood mononuclear cells (PMBCs)
	PMBCs
	Blood
	Serum
	Plasma
	Maternal blood
